# Supplementary material for: Parkin regulates adiposity by coordinating mitophagy with mitochondrial biogenesis in white adipocytes
Source: Nat Commun. 2022 Nov 4;13:6661. doi: 10.1038/s41467-022-34468-2 (PMC9636263; doi:10.1038/s41467-022-34468-2)

Supplementary Information

Parkin Regulates Adiposity by Coordinating Mitophagy with Mitochondrial Biogenesis in White Adipocytes

Timothy M. Moore et al.

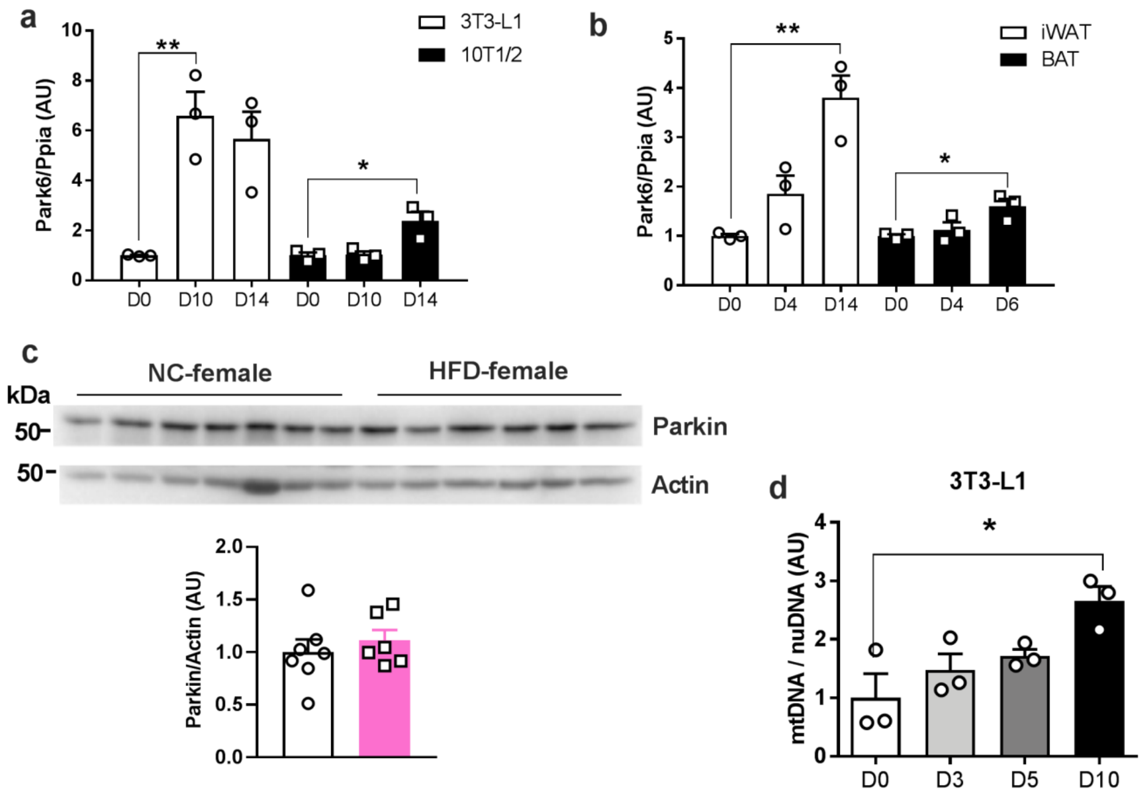

Suppl. Fig. 1: Parkin expression during the differentiation of primary adipocytes and 3T3-L1 preadipocytes.

(a) mRNA level of *Park6* during the differentiation of 3T3-L1 and 10T1/2 adipocytes (n=3 biological replicate) [Unpaired Student's t-test two-tailed. For 3T3-L1 D0 vs D10, *p*=0.0046, 95%CI=2.883 to 8.287, R squared=0.8917; for 10T1/2 D0 vs D14, *p*=0.0245, 95%CI=0.2918 to 2.471, R squared=0.7560]; (b) primary adipocytes from iWAT and BAT (n=3 biological replicate) [Unpaired Student's t-test two-tailed. For iWAT D0 vs D14, *p*=0.0035, 95%CI=1.540 to 4.063, R squared=0.9048; for BAT D0 vs D6, *p*=0.0185, 95%CI=0.1657 to 1.035, R squared=0.7861]. (c) Western blot analysis of Parkin in the gonadal white adipose tissues of NC vs. HFD-fed female mice (n=6-7 mice per diet). (d) mtDNA content of 3T3-L1 preadipocytes at day 0, day 3, day 5, and day 10 of the differentiation (n=3 biological replicate) [Unpaired Student's t-test two-tailed. For D0 vs D10, *p*=0.0263, 95%CI=0.3179 to 2.987, R squared=0.7471]. Data are presented as mean ± SEM. AU=arbitrary units. * $p < 0.05$, ** $p < 0.01$.

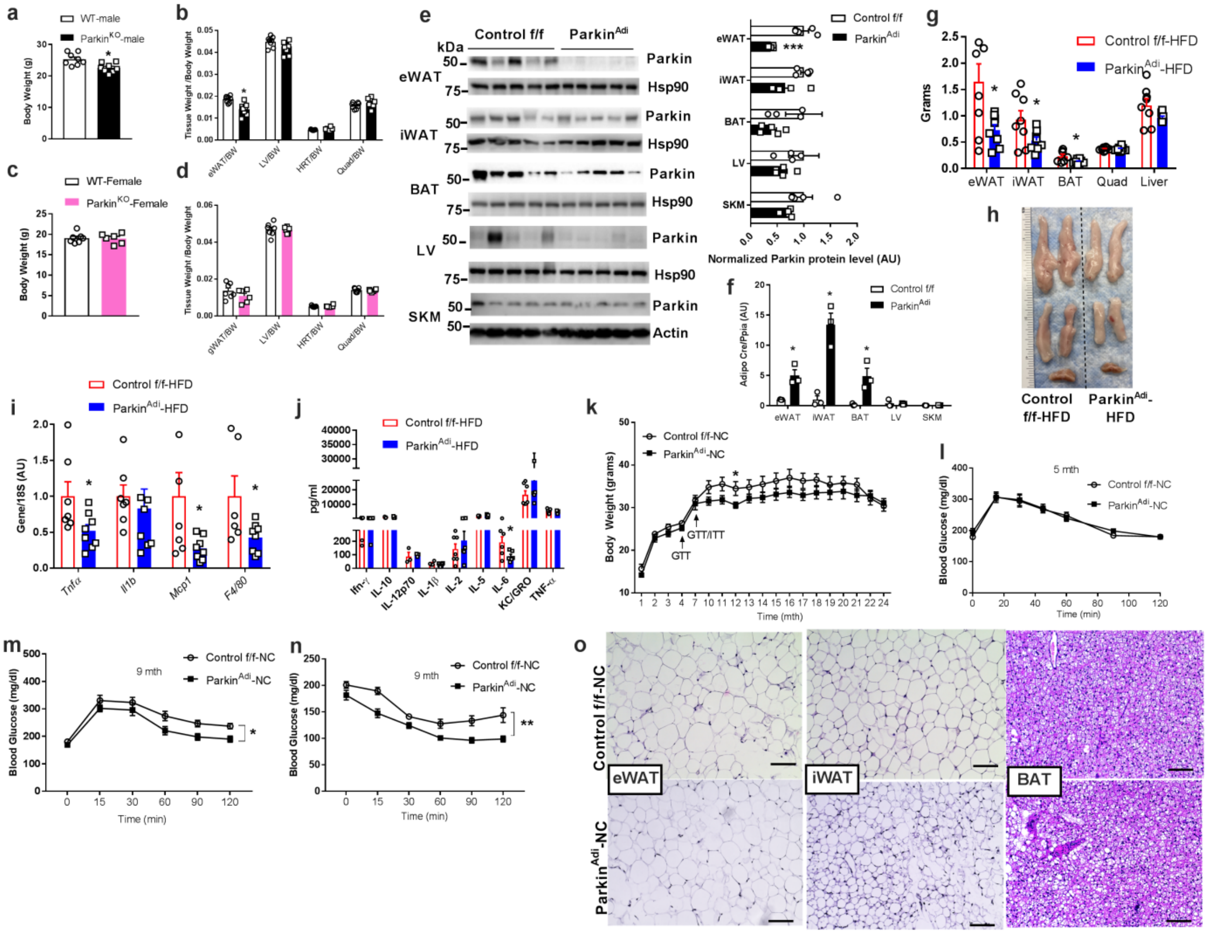

Suppl. Fig. 2: Body composition and histology of Parkin knockout mice.
(a and b) Body weight and body composition of NC-fed male WT and whole-body Parkin knockout (Parkin^{KO}) mice (n=6 mice per genotype) [Unpaired Student's t-test two-tailed. For WT-male vs Parkin^{KO}-male body weight, *p*=0.0058, 95%CI= -4.594 to -0.9435, R squared=0.4306; for WT-male vs Parkin^{KO}-male body composition eWAT/BW, *p*=0.0011, 95%CI= -0.006816 to -0.002141, R squared=0.5467]. (c and d) Body weight and body composition of NC-fed female WT and whole-body Parkin knockout mice (c: n=6 mice per genotype; d: n=7 mice for WT mice group, n=6 mice for Parkin^{KO} group). (e) Western blots analysis of Parkin in the eWAT, iWAT, BAT, liver (LV), and skeletal muscle (SKM) of Control f/f vs. Parkin^{Adi} mice fed with NC (n=5 mice per genotype) [Unpaired Student's t-test two-tailed. For Control f/f vs Parkin^{Adi} eWAT, *p*=<0.0001, 95%CI=0.4564 to 0.7868, R squared=0.8755]. (f) Adiponectin Cre expression in the eWAT, iWAT, BAT, LV, and SKM of male Control f/f and Parkin^{Adi} mice (n=3 mice per genotype) [Unpaired Student's t-test two-tailed. For Control f/f vs Parkin^{Adi} eWAT, *p*=0.0148, 95%CI=1.289 to 6.685, R squared=0.8080; for iWAT, p=0.0039, 95%CI=6.644 to 18.12, R squared=0.8998; for BAT, *p*=0.0245, 95%CI=0.9893 to 8.387, R squared=0.7558]. (g) Tissue weight of HFD-fed Control f/f and Parkin^{Adi} mice (n=8 mice per genotype) [Unpaired Student's t-test two-tailed. For Control f/f-HFD vs Parkin^{Adi}-HFD eWAT, *p*=0.0216, 95%CI= -1.685 to -0.1564, R squared=0.3229; for iWAT, *p*=0.0497, 95%CI=-0.7942 to -0.0006610, R squared=0.2479; for BAT, *p*=0.0288, 95%CI=-0.1906 to -0.01223, R squared=0.3171]. (h) Images of adipose tissues. (i) mRNA levels of inflammatory genes in eWAT of HFD-fed Control f/f and Parkin^{Adi} mice (n=7 mice for Control f/f group, except one sample in Control f/f group with undetectable *F4/80* gene expression, n=8 mice for Parkin^{Adi} group) [Unpaired Student's t-test two-tailed. For *Tnfα*, *p*=0.0395, 95%CI=-

0.9359 to -0.02693, R squared=0.2871; for *Mcp1*, *p*=0.0387, 95%CI=-1.401 to -0.04362, R squared=0.2891; for *F4/80*, *p*=0.0466, 95%CI=-1.132 to -0.01025, R squared=0.2909]. (j) The levels of inflammatory cytokines and chemokines in the plasma of HFD-fed Control f/f and $\text{Parkin}^{\text{Adi}}$ mice (n=7 mice per genotype, exception made for samples with undetectable cytokines and chemokines) [Unpaired Student's t-test two-tailed. For IL-6, *p*=0.0376, 95%CI=-200.0 to -7.249, R squared=0.3647]. (k) Body weight of NC-fed male Control f/f and $\text{Parkin}^{\text{Adi}}$ mice (n=7 mice per genotype) [Unpaired Student's t-test two-tailed. For 12 months Control f/f-NC vs $\text{Parkin}^{\text{Adi}}$-NC, *p*=0.0489, 95%CI-7.770 to -0.02175, R squared=0.2857]. (l, m, and n) Glucose tolerance test and insulin tolerance test performed on 5 months old and 9 months old NC-fed Control f/f and $\text{Parkin}^{\text{Adi}}$ mice (l: n=7 mice for Control f/f group, n=5 mice for $\text{Parkin}^{\text{Adi}}$ group per genotype; m: n=7 mice for Control f/f group, n=6 mice for $\text{Parkin}^{\text{Adi}}$ group per genotype [Two-way ANOVA. F(1,11)=5.048, *p*=0.0461, 95%CI=0.7351 to 71.27]; n: n=7 mice for Control f/f group, n=6 mice for $\text{Parkin}^{\text{Adi}}$ group [Two-way ANOVA. F(1,11)=11.29, *p*=0.0064, 95%CI=10.76 to 51.61]); 90 min *p*=0.0156, 95%CI=-85.94 to -11.16, R squared=0.4261; 120 min, *p*=0.0086, 95%CI=-79.86 to -14.67, R squared=0.4807. Two-way ANOVA. For Control f/f-NC vs $\text{Parkin}^{\text{Adi}}$-NC, p<0.0001, F(1,35)=54.84]. (o) H&E-stained sections of eWAT, iWAT, and BAT from NC-fed mice at 6 months old; scale bars = 50 µm. Data are presented as mean ± SEM. AU=arbitrary units. * *p* < 0.05, ** *p* < 0.01.

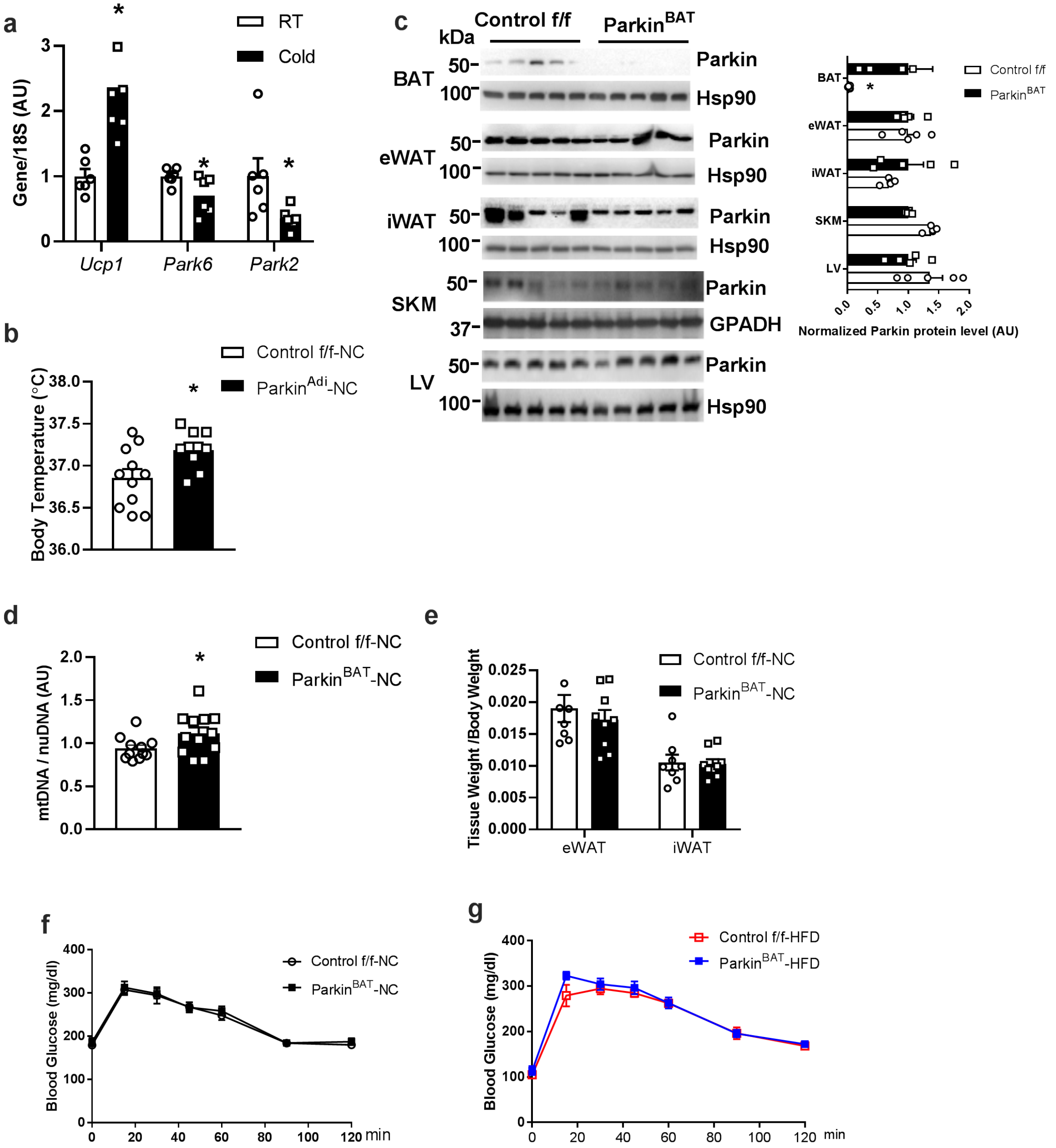

Suppl. Fig. 3: Brown adipose-specific Parkin deletion does not impact glucose homeostasis. (a) mRNA levels of *Ucp1*, *Park6*, and *Park2* genes in the BAT of male mice housed in room temperature (RT) or cold room for 4 hours (4°C) (n=6 mice for RT group, n=7 mice for Cold group, except one sample in Cold group with undetectable *Park6* and *Park2* gene expression) [Unpaired Student's t-test two tailed. For *Ucp1*, *p*=0.0020, 95%CI=0.6222 to 2.114, R squared=0.5969; for *Park6*, *p*=0.0443, 95%CI=-0.5816 to -0.009134, R squared=0.3458; for *Park2*, *p*=0.0452, 95%CI=-1.277 to -0.01681, R squared=0.3435]. (b) Body temperature of 2 months old NC-fed Control f/f and $\text{Parkin}^{\text{Adi}}$ mice (n=11 mice for Control f/f group, n=8 mice for $\text{Parkin}^{\text{BAT}}$ group) [Unpaired Student's t-test two tailed. For Control f/f-NC vs $\text{Parkin}^{\text{Adi}}$-NC, *p*=0.0356, 95%CI=0.02514 to 0.6408, R squared=0.2345]. (c) Western blots analysis of Parkin

in the BAT, eWAT, iWAT, SKM, and LV of Control f/f and $Parkin^{BAT}$ mice (n=5 per genotype). [Unpaired Student's t-test two tailed. For Control f/f vs $Parkin^{BAT}$ BAT, p=0.0421, 95%CI=0.04405 to 1.892, R squared=0.4218]. (d) mtDNA content of the BAT from Control f/f and $Parkin^{BAT}$ mice (n=11 for Control f/f group, n=12 mice for $Parkin^{BAT}$ group) [Unpaired Student's t-test two tailed. For Control f/f-NC vs $Parkin^{BAT}$-NC, p=0.0430, 95%CI=0.006149 to 0.3473, R squared=0.1810]. (e) eWAT and iWAT tissue weight of Control f/f and $Parkin^{BAT}$ mice (n=8 mice for Control f/f group, n=9 mice for $Parkin^{BAT}$ group). (f and g) Glucose tolerance analysis of NC-fed and HFD-fed (5 weeks) Control f/f and $Parkin^{BAT}$ mice (f: n=7 mice for Control f/f-NC group, n=4 mice for $Parkin^{BAT}$-NC group; g: n=5 mice for Control f/f-HFD group, n=4 mice for $Parkin^{BAT}$-HFD group). Data are presented as mean ± SEM. AU=arbitrary units. * $p < 0.05$.

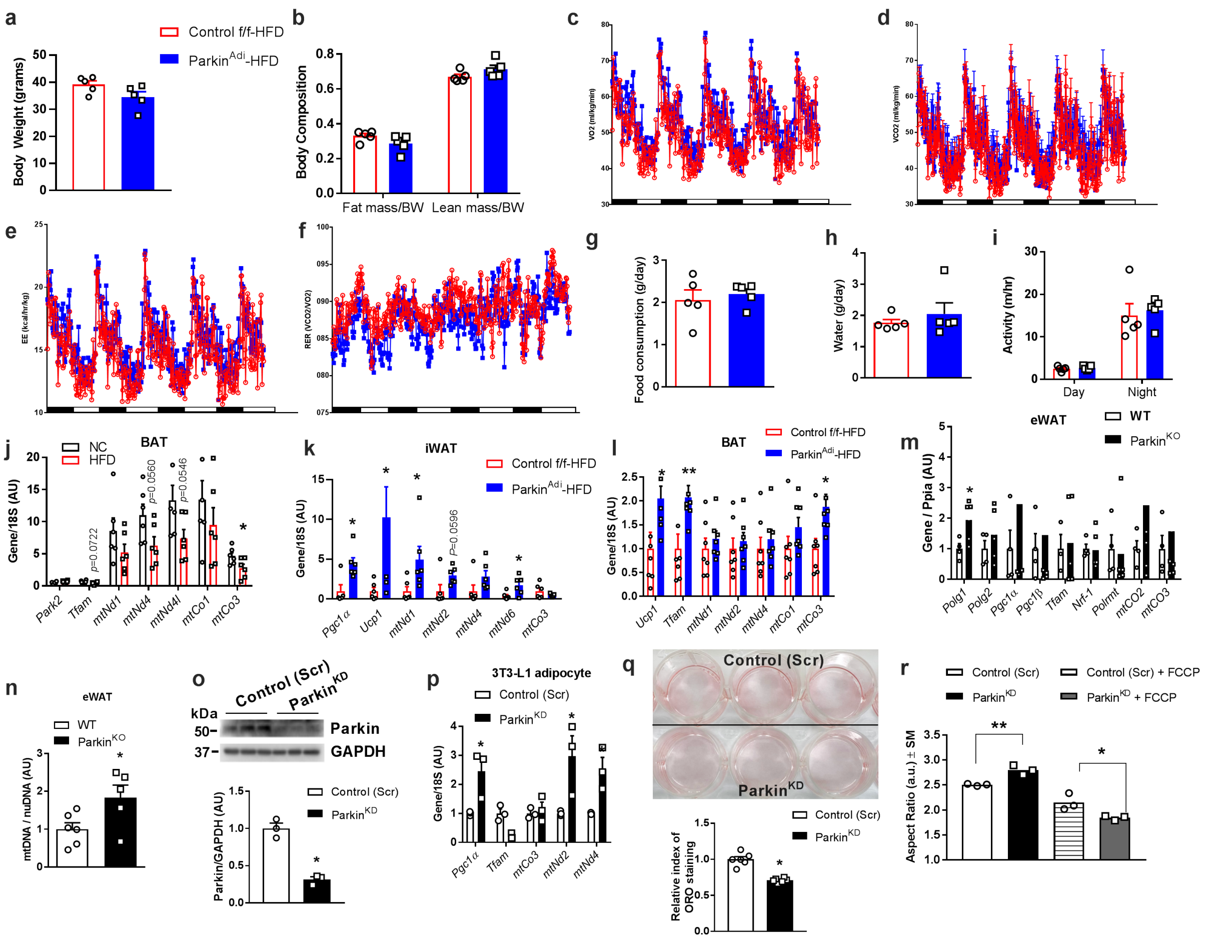

Suppl. Fig. 4: Parkin deletion elevates energy expenditure and mitochondrial activity in adipocytes.

(a and b) Body weight and body composition of 4 weeks of HFD-fed Control f/f and Parkin^{Adi} mice that housed in the chamber (n=5 mice per genotype). (c, d, e, and f) recording oxygen consumption, CO2 production, EE, and RER of HFD-fed Control f/f and Parkin^{Adi} mice for 4 days (n=5 mice per genotype). (g, h, and i) Food consumption per day, water consumption per day, and locomotor activity in day and night of HFD-fed Control f/f and Parkin^{Adi} mice (n=5 mice per genotype). (j, k, l, and m) mRNA levels of mitochondrial and related genes in the BAT of mice fed with NC and HFD (n=6 mice per genotype) [Unpaired Student's t-test two tailed. For NC vs HFD *Tfam*, *p*=0.0722, 95%CI= -0.4984 to 0.02564, R squared=0.2878; for *mtNd4*, p=0.0560, 95%CI=-9.549 to 0.1457, R squared=0.3184; for *mtNd4l*, *p*=0.0546, 95%CI=-12.01 to 0.1427, R squared=0.3213; for *mtCo3*, *p*=0.0446, 95%CI= -3.717 to -0.05487, R squared=0.3450], in the BAT of Control f/f and Parkin^{Adi} mice fed with HFD (n=7 mice per genotype) [Unpaired Student's t-test two tailed. For HFD-fed Control f/f vs Parkin^{Adi} *Ucp1*, *p*=0.0309, 95%CI=0.1140 to 1.982, R squared=0.3325; for *Tfam*, *p*=0.0184, 95%CI=0.2158 to 1.929, R squared=0.3827; for *mtCo3*, *p*=0.0233, 95%CI=0.1415 to 1.612, R squared=0.3600], in the iWAT of HFD-fed Control f/f and Parkin^{Adi} mice (n=6 mice per genotype) [Unpaired Student's t-test two tailed. For HFD-fed Control f/f vs Parkin^{Adi} *Pgc1α*, *p*=0.0121, 95%CI=0.9530 to 5.917, R squared=0.5213; for *Ucp1*, *p*=0.0360, 95%CI=0.7373 to 17.82, R squared=0.3694; for *mtNd1*, *p*=0.0472, 95%CI=0.05842 to 7.861, R squared=0.3384; for *mtNd6*, *p*=0.0235, 95%CI=0.2245 to 2.489, R squared=0.4162], in the eWAT of WT and Parkin^{KO} mice fed with NC (n=4 mice for WT group, except one sample in

WT group with undetectable *Tfam* gene expression, n=5 mice for Parkin^{KO} group) [Unpaired Student's t-test two tailed. For NC-fed WT vs Parkin^{KO} *Polg1*, *p*=0.0423, 95%CI=0.04330 to 1.839, R squared=0.4674]. (n) mtDNA content in the eWAT of WT and Parkin^{KO} mice (n=5 or 6 mice per genotype) [Unpaired Student's t-test two tailed. For WT vs Parkin^{KO}, p=0.0431, 95%CI=0.03202 to 1.633, R squared=0.3808]. (o) Western blot and densitometry analysis of Parkin (normalized to GAPDH) in 3T3-L1 Control (Scr) and Parkin^{KD} adipocytes. (n=3 biological replicate) [Unpaired Student's t-test two tailed. For 3T3-L1 Control (Scr) vs Parkin^{KD} adipocytes, *p*=0.0011, 95%CI=-0.9115 to -0.4585, R squared=0.9463]. (p) mRNA levels of mitochondrial genes in 3T3-L1 Control (Scr) and Parkin^{KD} adipocytes (n=3 biological replicate). [Unpaired Student's t-test two tailed. For 3T3-L1 Control (Scr) vs Parkin^{KD} *Pgc1α*, *p*=0.0366, 95%CI=0.1469 to 2.757, R squared=0.7046; *mtNd2*, *p*=0.0495, 95%CI=0.006640 to 3.929, R squared=0.6599; *mtNd4*, *p*=0.0215, 95%CI=0.3666 to 2.663, R squared=0.7703]. (q) Oil O Red staining and the quantification analysis of ORO in the differentiated Control (Scr) and Parkin^{KD} adipocytes (n=3 biological replicate) [Unpaired Student's t-test two tailed. For Control (Scr) vs Parkin^{KD} adipocytes, *p*<0.0001, 95%CI=-0.3779 to -0.2053, R squared=0.8501]. (r) Mitochondrial aspect ratio analysis of Control (Scr) and Parkin^{KD} adipocytes (n=3 biological replicate) [Unpaired Student's t-test two tailed. For Control (Scr) vs Parkin^{KD}, p=0.0061, 95%CI=0.1396 to 0.4468, R squared=0.8754; for Control (Scr) + FCCP vs Parkin^{KD} + FCCP, *p*=0.0385, 95%CI=-0.5822 to -0.02604, R squared=0.6974]. Data are presented as mean ± SEM. AU=arbitrary units. * $p < 0.05$, ** $p < 0.01$.

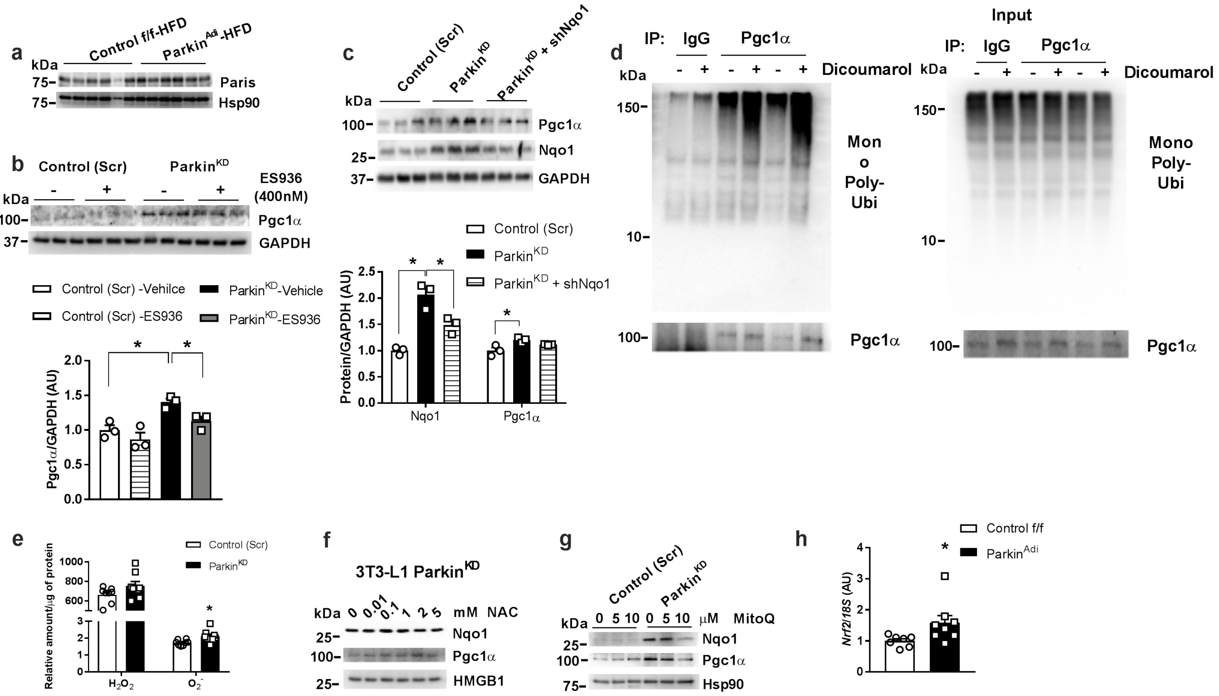

Suppl. Fig. 5: Nqo1 preserves Pgc1α protein in adipocytes.

(a) Western blot analysis of Paris in eWAT of Control f/f and Parkin^{Adi} mice fed with HFD (n=6 mice per genotype). (b and c) Western blot and densitometry analysis of Pgc1α and Nqo1 (normalized to GAPDH) in the differentiated 3T3-L1 Control (Scr) and Parkin^{KD} adipocytes treated with vehicle or ES936 (Nqo1 inhibitor) [Unpaired Student's t-test two tailed. For Control (Scr)-Vehicle vs Parkin^{KD}-Vehicle, p=0.0093, 95%CI=0.1655 to 0.6437, R squared=0.8466; for Parkin^{KD}-Vehicle vs Parkin^{KD}-ES936, p=0.0291, 95%CI=-0.5084 to -0.04601, R squared=0.7348] and in 3T3-L1 Control (Scr), Parkin^{KD,} and Parkin^{KD} + shNqo1 3T3-L1 preadipocytes (n=3 biological replicate) [Unpaired Student's t-test two tailed. For Nqo1 Control (Scr) vs Parkin^{KD}, p=0.0020, 95%CI=0.6534 to 1.477, R squared=0.9280; for Nqo1 Parkin^{KD} vs Parkin^{KD} + shNqo1, p=0.0261, 95%CI=-1.047 to -0.1129, R squared=0.7482; for Pgc1α Control (Scr) vs Parkin^{KD}, p=0.0364, 95%CI=0.02088 to 0.3863, R squared=0.7052]. (d) Extra co-immunoprecipitation of IgG (negative control) and Pgc1α in differentiated 3T3-L1 Control (Scr) and Parkin^{KD} adipocytes treated with vehicle or Dicoumarol. (e) Relative amount of H_2O_2 and O_2^- in differentiated adipocytes of Control (Scr) and Parkin^{KD} (n=8 biological replicate) [Unpaired Student's t-test two tailed. For, O_2^- p=0.0162, 95%CI=0.08281 to 0.6880, R squared=0.3477]. (f and g) Western blot analysis of Pgc1α and Nqo1 in the indicated concentration of NAC or MitoQ treated 3T3-L1 adipocytes. (h) mRNA level of *Nrf2* in the eWAT of HFD-fed Control f/f and Parkin^{Adi} mice (n=7 mice for Control f/f group, n=8 mice for Parkin^{Adi} group) [Unpaired Student's t-test two tailed. For Control f/f vs Parkin^{Adi}, p=0.0447, 95%CI=0.01597 to 1.145, R squared=0.2752]. Data are presented as mean ± SEM. AU=arbitrary units. * $p < 0.05$.

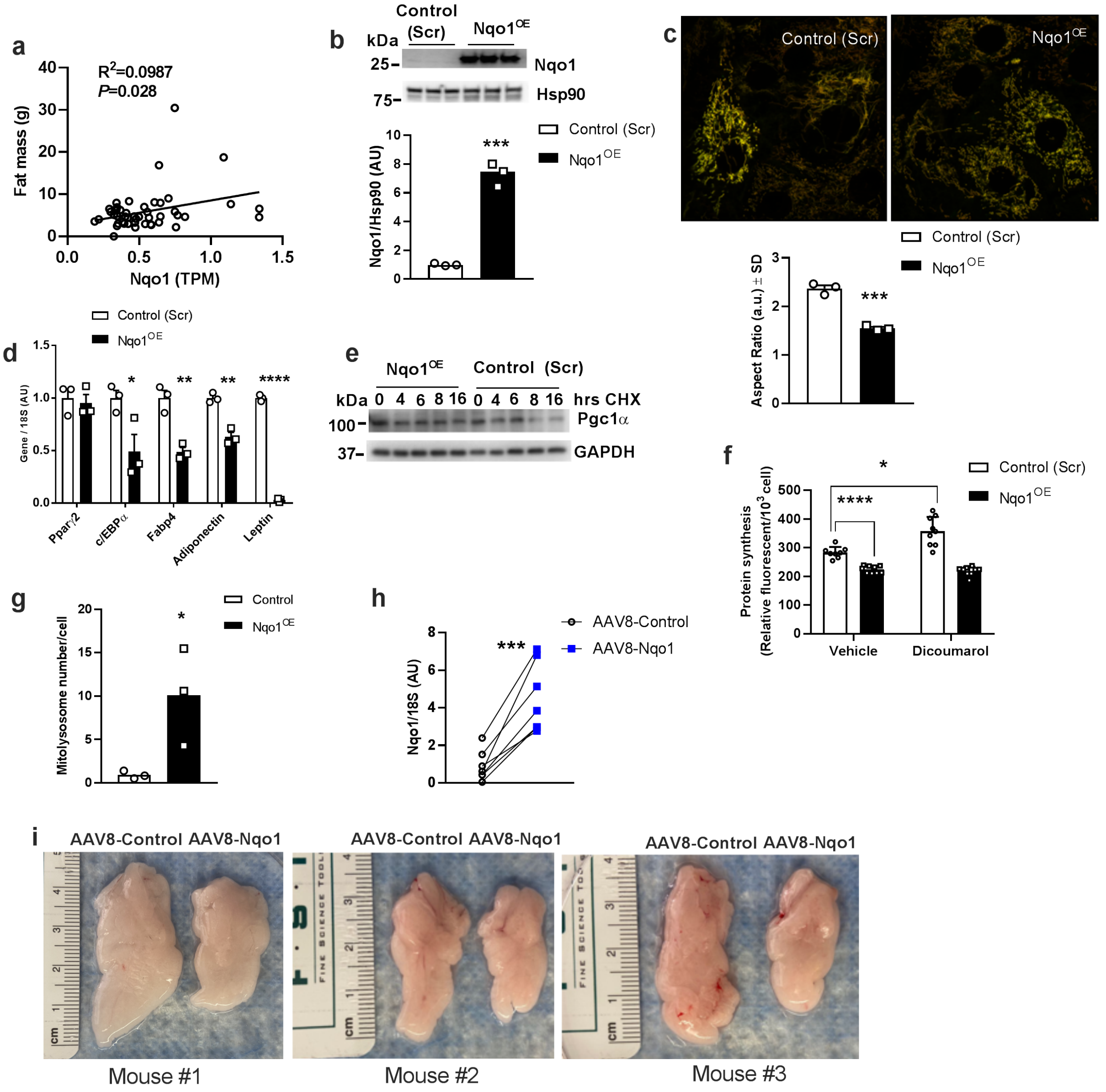

Suppl. Fig. 6: Overexpression of Nqo1 enhances mitochondrial biogenesis *in vitro* and *in vivo*.

(a) *Nqo1* gene expression in adipose tissue from 49 strains of mice (4 mice per strain) versus total fat mass (g) [Linear regression, F=5.144, *p*=0.0280, 95%CI=0.6623 to 11.05, R squared=0.09866]. (b) Western blot and densitometry analysis of Nqo1 protein level (normalized to Hsp90) in the differentiated 3T3-L1 Control (Scr) and Nqo1^{OE} adipocytes (n=3 biological replicate) [Unpaired Student's t-test two-tailed, *p*=0.0002, 95%CI=5.006 to 7.598, R squared=0.9785]. (c) Mitochondrial morphology of the differentiated 3T3-L1 Control (Scr) and Nqo1^{OE} adipocytes. The bar graph at the bottom is the normalized aspect ratio (n=3 biological replicate) [Unpaired Student's t-test two-tailed, *p*=0.0004, 95%CI=-1.023 to -0.6150, R squared=0.9688]. (d) mRNA of adipogenesis genes: *Pparr2*, *C/Ebpα*, *Fabp4*, *Adiponectin*, and *Leptin* in the differentiated 3T3-L1 Control (Scr) and Nqo1^{OE} adipocytes (n=3 biological replicate) [Unpaired Student's t-test two-tailed, for *c/EBPα*, *p*=0.0456, 95%CI=-0.9969 to -0.01609, R squared=0.6727; for *Fabp4*, *p*=0.0037,

95%CI=-0.7470 to -0.2779, R squared=0.9019; for *Adiponectin*, *p*=0.0019, 95%CI=-0.5104 to -0.2280, R squared=0.9295; for *Leptin*, *p*<0.0001, 95%CI=-1.028 to -0.9156, R squared=0.9983]. (e) Western blot analysis of Pgc1α in the differentiated 3T3-L1 Control (Scr) and $Nqo1^{OE}$ adipocytes treated with 100 μg/ml CHX at the indicated time. (f) The protein synthesis rate of the differentiated 3T3-L1 Control (Scr) and $Nqo1^{OE}$ adipocytes treated with vehicle or Dicoumarol (n=9 for each group, except one sample in Control (Scr) vehicle with undetectable value) [Unpaired Student's t-test two-tailed, for vehicle Control (Scr) vs $Nqo1^{OE}$, *p*<0.0001, 95%CI=-75.44 to -41.84, R squared=0.7867; for Control (Scr)-vehicle vs Control (Scr)-Dicoumarol, *p*=0.0015, 95%CI=33.33 to 114.7, R squared=0.5005]. (g) Normalized mitolysosome number in the differentiated 3T3-L1 Control (Scr) and $Nqo1^{OE}$ adipocytes (n=3 biological replicate) [Unpaired Student's t-test two-tailed, *p*=0.0473, 95%CI=0.1758 to 18.24, R squared=0.6670]. (h) mRNA level of Nqo1 in the eWAT of AAV8 injected mice (n=7 mice per group) [Paired Student's t-test two-tailed, t=6.612. *p*=0.0006, 95%CI=2.276 to 4.951, R squared=0.8793]. (i) Image of the eWAT of AAV8-Control or AAV8-Nqo1 injected mice. Data are presented as mean ± SEM, except (c) is presented as mean ± SD. AU=arbitrary units. * $p < 0.05$, ** $p < 0.01$, *** $p < 0.001$, **** $p < 0.0001$.

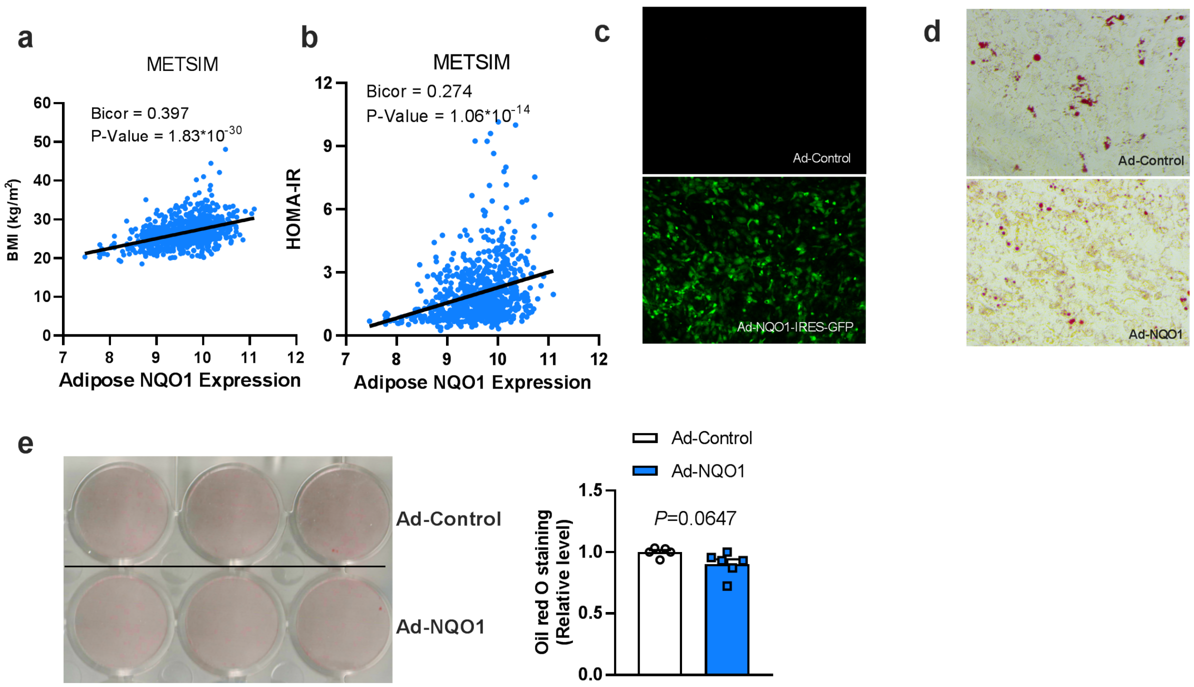

Suppl. Fig. 7: Overexpression of NQO1 in human SubQ adipocytes elevates mtDNA content and improves mitochondrial function.

(a and b) The association of human subcutaneous adipose NQO1 gene expression with BMI and HOMA-IR from METSIM study (n=770) [Linear regression, for adipose NQO1 and BMI association, F=157.5, *p*<0.0001, 95%CI=2.121 to 2.908, R squared=0.1702; for adipose NQO1 and HOMA-IR association, F=75.64, *p*<0.0001, 95%CI=0.5612 to 0.8883, R squared=0.08976]. (c) Validating NQO1 overexpression in ad-Control and ad-NQO1 treated primary human subcutaneous adipocytes (n=3 for Ad-Control group, n=5 for Ad-NQO1 group). (d and e) Oil Red O staining of ad-Control and ad-NQO1 treated primary human subcutaneous adipocytes. The bar graph on the right is the densitometry of oil red o staining in the adipocytes (n=5 for Ad-Control group, n=6 for Ad-NQO1 group) [Unpaired Student's t-test two-tailed]. Data are presented as mean ± SEM.

Table S1. Primer list

Species	Gene	Forward	Reverse
Mouse	18S	CGCCGCTAGAGGTGAAATTCT	CGAACCTCCGACTTTCGTTCT
	Acc1	CTGAAGCAGATCCGCAGCTT	GGTGAGATGTGCTGGGTCATG
	Adipo Cre	AGGTGTAGAGAAGGCACTTAG	CTAATCGCCATCTTCCAGCAGG
	Adiponectin	TGTTGGAATGACAGGAGCTGAA	CACACTGAAGCCTGAGCGATAC
	c/Ebpa	CCCCCACTCAGCTTACAACAGG	CACCCCACAAAGCCCAGAAAC
	Cd36	TCCAGCCAATGCCTTTGC	TGGAGATTACTTTTTCAGTGCAGAA
	Cpt1a	CCAGGCTACAGTGGGACATT	GAACTTGCCCATGTCCTTGT
	Erra	GGAGGACGGCAGAAGTACAAA	GCGACACCAGAGCGTTCAC
	F4/80	TTTGGCTATGGGCTTCCAGTC	GCAAGGAGGACAGAGTTTATCGTG
	Fabp4	TTCGATGAAATCACCGCAGA	GGTCGACTTTCCATCCCACTT
	Fas	TGCTCCCAGCTGCAGGC	GCCCGGTAGCTCTGGGTGTA
	Il1b	GACGGCACACCCACCCT	AAACCGTTTTTCCATCTTCTTT
	Leptin	GAGACCCCTGTGTCGGTTC	CTGCGTGTGTGAAATGTCATTG
	Mcp1	CAGCCAGATGCAGTTAACGC	GCTGCTGGTGATCCTCTTG
	mtCo1	TCCAACTCATCCCTTGACATC	TCCTGCTATGATAGCAAACACT
	mtCo3	GCAGGATTCTTCTGAGCGTTCT	GTCAGCAGCCTCCTAGATCATGT
	mtNd1	GTTGGTCCATACGGCATTTT	TGGGTGTGGTATTGGTAGGG
	mtNd2	CGCCCCATTCCACTTCTGATT	TTAAGTCCTCCTCATGCCCCT
	mtNd4	GCCTGATTACTGCCACTAATA	GGTTCCCTCATCGGGTAATAA
	mtNd4l	ACCTCACCATAGCCTTCTCAC	TAGTCCTACAGCTGCTTCGC
	mtNd6	ACAACTATATATTGCCGC	GATATACGACTGCTATAGCTA
	Mttp	CGTGGTGAAAGGGCTTATTC	TCGCGATACCACAGACTGAA
	Nqo1	TTCTCTGGCCGATTCAGAGT	GGCTGCTTGGAGCAAAATAG
	Nrf1	GAA CTG CCA ACC ACA GTC AC	CGT CTG GAT GGT CAT TTC AC
	Nrf2	CTCGCTGGAAAAAGAAGTGG	CGGTCCAGGAGTTCAGAGAG
	Park2	GGAAGCCATAGCTGGAGTTG	AAACCTGACAGAAACGCTGG
	Park6	GGATGTCGTCCTGAAGGGAG	GCTTCGCTGGAGGAACCTG
	Polg1	TAGCTGGCTGGTCCAAGAGT	CGACGTGGAGGTCTGCTT
	Polg2	CCGTTTTCCAGCGTAGTCTC	TTCTGTGTGGCCTGGCTATT
	Polrmt	CTCATCTCAGGTGTGCCCTC	TCTGCAGCTCAAGAAGGAGC
	Ppara	CCTGAACATCGAGTGTCGAATAT	GGTCTTCTTCTGAATCTTGCAGCT
	Pparg2	TCA CAA GAG CTG ACC CAA TGG	GGT TCT ACT TTG ATC GCA CTT TG
	Ppargc1a	TGAGGACCGCTAGCAAGTTT	TGAAGTGGTGTAGCGACCAA
	Ppargc1b	CTGAGTCAAAGTCACTGGCG	GCTCTCGTCCTTCTTCCTCA
	Scd1	TGGGTTGGCTGCTTGTG	GCGTGGGCAGGATGAAG
	Srebp1c	GCTTCCAGAGAGGAGCCCAG	GGAGCCATGGATTGCACATT

	Tfam	AGC TTG TAA ATG AGG CTT GGA	AGA TGT CTC CGG ATC GTT TC
	Tnfa	CAC AAG ATG CTG GGA CAG TGA	TCC TTG ATG GTG GTG CAT GA
	Ucp1	GGAAAGGGACGACCCCTAATC	CCGGCAACAAGAGCTGACA
Human	ACTIN	GATGAGATTGGCATGGCTTT	GTCACCTTCACCGTTCCAGT
	B2M	TGCTGTCTCCATGTTTGATGTATCT	TCTCTGCTCCCCACCTCTAAGT
	c/EBPa	CTAGAGATCTGGCTGTGGGG	TCATAACTCCGGTCCCTCTG
	FASN	CTGGCTCAGACACTCTATCC	CAGGTTGTCCCTGTGATCCT
	LPER	AGGACGAAAGCCAGAGACAA	AAATGCCTGGGCCTCTATCT
	PPARa	ATGGCATCCAGAACAAGGAG	TCCCGTCTTTGTTCATCACA
	PPARg	GCTGTGCAGGAGATCACAGA	GGGCTCCATAAAGTCACCAA
	tRNA-Leu	CACCCAAGAACAGGGTTTGT	TGGCCATGGGTATGTTGTTA

Original gel blots of Supplementary Figures

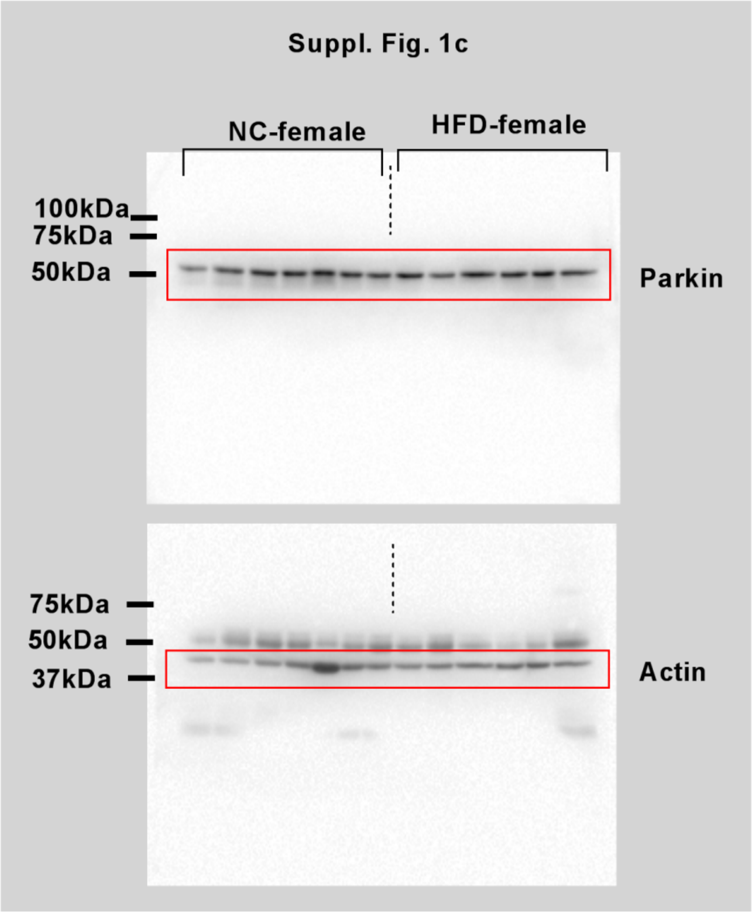

Suppl. Fig. 2e

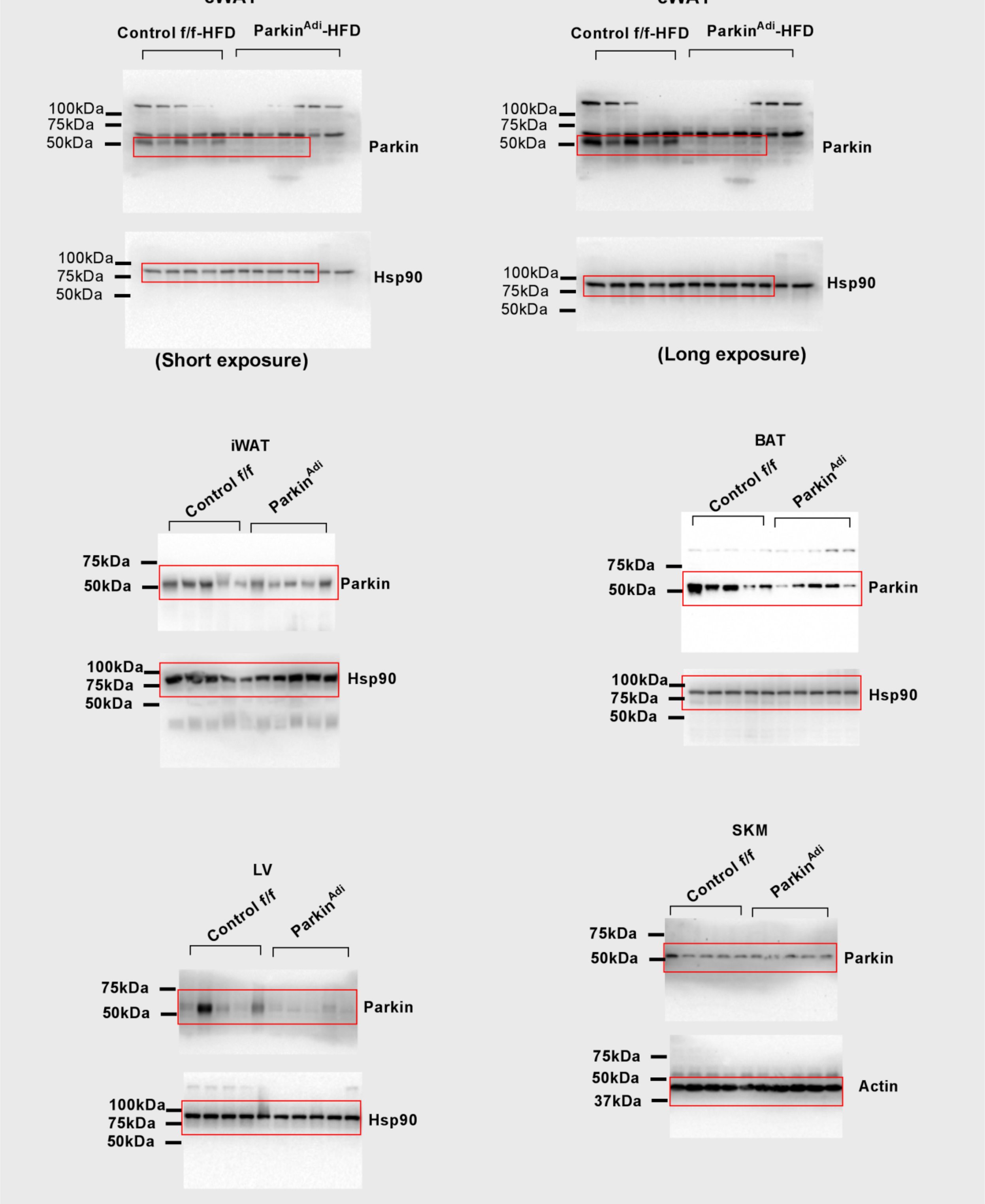
eWAT
Control f/f-HFD
ParkinAdi-HFD
100kDa
75kDa
50kDa
Parkin
Hsp90
(Short exposure)
eWAT
Control f/f-HFD
ParkinAdi-HFD
100kDa
75kDa
50kDa
Parkin
Hsp90
(Long exposure)
iWAT
Control f/f
ParkinAdi
75kDa
50kDa
Parkin
100kDa
75kDa
50kDa
Hsp90
BAT
Control f/f
ParkinAdi
75kDa
50kDa
Parkin
100kDa
75kDa
50kDa
Hsp90
LV
Control f/f
ParkinAdi
75kDa
50kDa
Parkin
100kDa
75kDa
50kDa
Hsp90
SKM
Control f/f
ParkinAdi
75kDa
50kDa
Parkin
75kDa
50kDa
37kDa
Actin

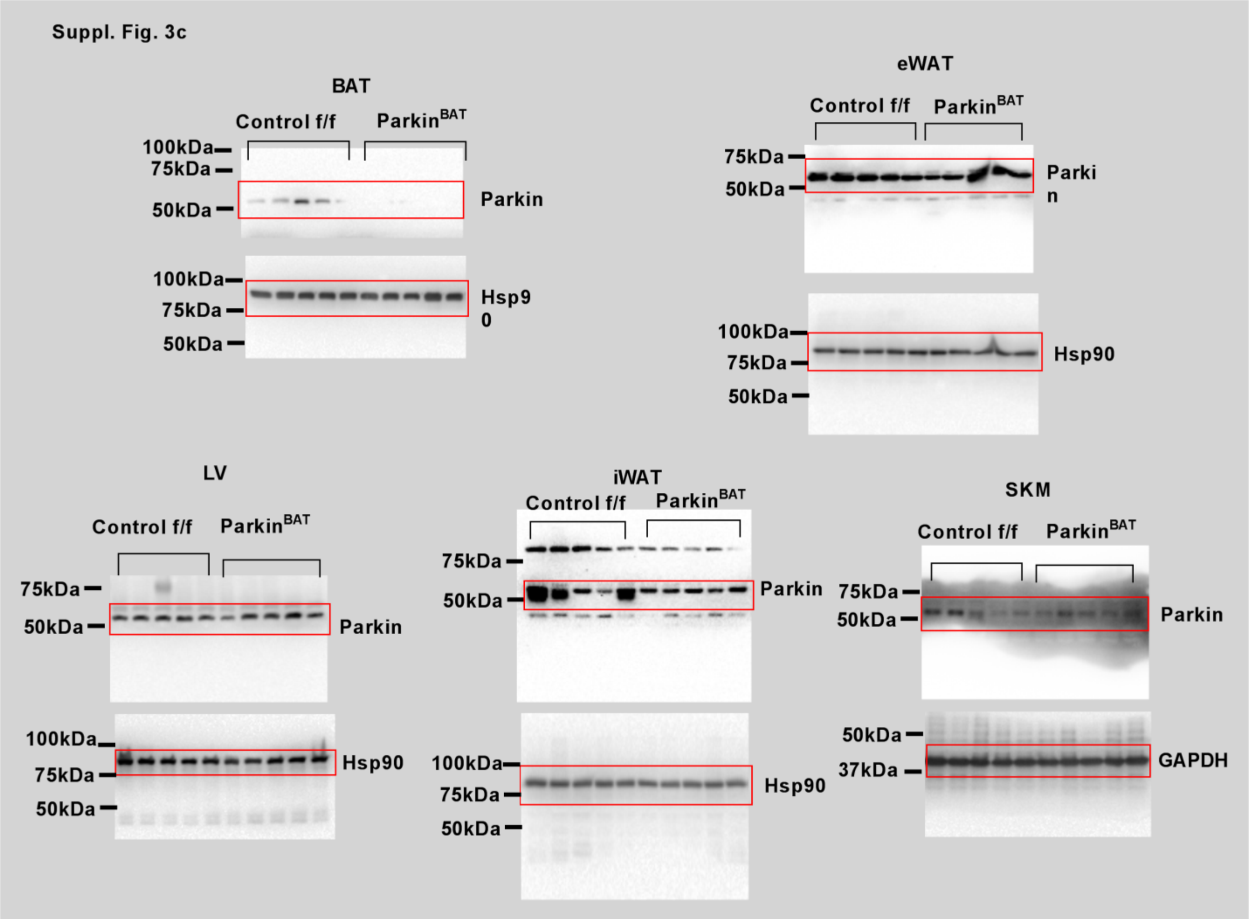
Suppl. Fig. 3c
BAT
Control f/f
Parkin^BAT
100kDa
75kDa
50kDa
Parkin
Hsp90
eWAT
Parkin
LV
iWAT
SKM
GAPDH
37kDa

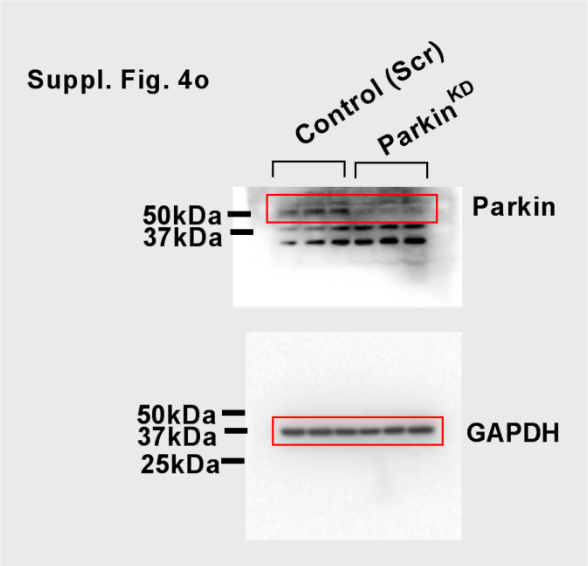
Suppl. Fig. 4o
Control (Scr)
Parkin^KD
50kDa
37kDa
25kDa
Parkin
GAPDH

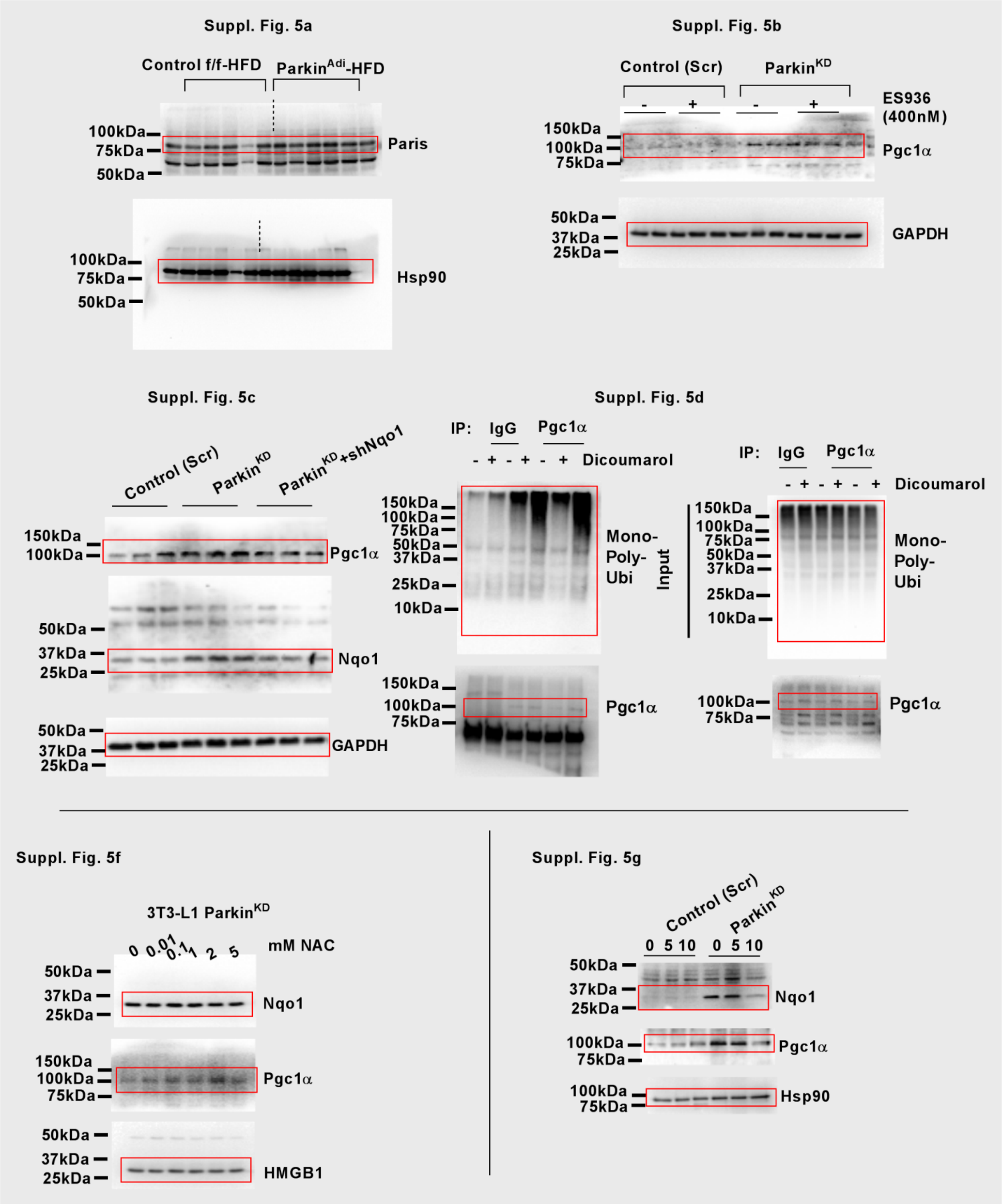

Suppl. Fig. 5a
Control f/f-HFD
ParkinAdi-HFD
100kDa
75kDa
50kDa
Paris
Hsp90
Suppl. Fig. 5b
Control (Scr)
ParkinKD
ES936 (400nM)
150kDa
100kDa
75kDa
Pgc1α
50kDa
37kDa
25kDa
GAPDH
Suppl. Fig. 5c
Control (Scr)
ParkinKD
ParkinKD+shNqo1
Pgc1α
Nqo1
GAPDH
Suppl. Fig. 5d
IP: IgG Pgc1α
Dicoumarol
10kDa
Mono-
Poly-
Ubi
Input
Pgc1α
Suppl. Fig. 5f
3T3-L1 ParkinKD
0 0.01 0.1 1 2 5
mM NAC
Nqo1
Pgc1α
HMGB1
Suppl. Fig. 5g
Control (Scr)
ParkinKD
0 5 10 0 5 10
Nqo1
Pgc1α
Hsp90

Suppl. Fig. 6b

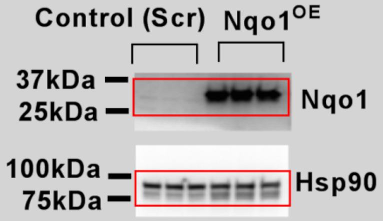

Suppl. Fig. 6e

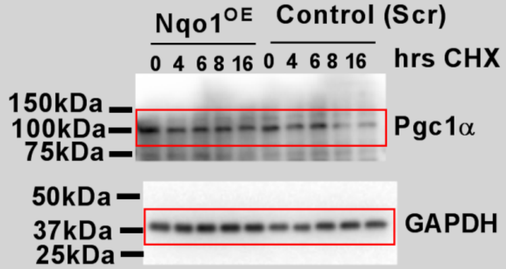

Supplement: Supplementary file 1 — Supplementary Information [file 41467_2022_34468_MOESM1_ESM.pdf]
