# Supplementary material for: Parkin regulates adiposity by coordinating mitophagy with mitochondrial biogenesis in white adipocytes
Source: Nat Commun. 2022 Nov 4;13:6661. doi: 10.1038/s41467-022-34468-2 (PMC9636263; doi:10.1038/s41467-022-34468-2)
Supplement: Supplementary file 3 — Reporting Summary [file 41467_2022_34468_MOESM3_ESM.pdf]

nature portfolio

Corresponding author(s): Zhenqi Zhou

Last updated by author(s): Jul 6, 2022

Reporting Summary

Nature Portfolio wishes to improve the reproducibility of the work that we publish. This form provides structure for consistency and transparency in reporting. For further information on Nature Portfolio policies, see our Editorial Policies and the Editorial Policy Checklist.

Statistics

For all statistical analyses, confirm that the following items are present in the figure legend, table legend, main text, or Methods section.

n/a	Confirmed	
☐	☒	The exact sample size (*n*) for each experimental group/condition, given as a discrete number and unit of measurement
☐	☒	A statement on whether measurements were taken from distinct samples or whether the same sample was measured repeatedly
☐	☒	The statistical test(s) used AND whether they are one- or two-sided<br>*Only common tests should be described solely by name; describe more complex techniques in the Methods section.*
☒	☐	A description of all covariates tested
☒	☐	A description of any assumptions or corrections, such as tests of normality and adjustment for multiple comparisons
☐	☒	A full description of the statistical parameters including central tendency (e.g. means) or other basic estimates (e.g. regression coefficient) AND variation (e.g. standard deviation) or associated estimates of uncertainty (e.g. confidence intervals)
☐	☒	For null hypothesis testing, the test statistic (e.g. *F*, *t*, *r*) with confidence intervals, effect sizes, degrees of freedom and *P* value noted<br>*Give P values as exact values whenever suitable.*
☒	☐	For Bayesian analysis, information on the choice of priors and Markov chain Monte Carlo settings
☒	☐	For hierarchical and complex designs, identification of the appropriate level for tests and full reporting of outcomes
☒	☐	Estimates of effect sizes (e.g. Cohen's *d*, Pearson's *r*), indicating how they were calculated

Our web collection on statistics for biologists contains articles on many of the points above.

Software and code

Policy information about availability of computer code

Data collection	qPCR: QuantStudio5; Western blot: BIO-RAD ChemiDoc XRS imaging system; Seahorse assay:XF96 Extracellular Flux Analyzer (Seahorse Biosciences); Body composition: Bruker Minispec; Fluoresence imaging: Zeiss LSM880 confocal microscope and Lionheart LX; Metabolic analysis: Promethion metabolic screening system (Sable Systems); Insulin, and leptin: Meso Scale Discovery; Blood glucose: HemoCue; RNAseq: Illumina HiSeq 3000.
Data analysis	Statistical significance was assessed using GraphPad Prism Version 9.4.1.<br>FIJI (NIH, 64-bit, 2.9.0)

For manuscripts utilizing custom algorithms or software that are central to the research but not yet described in published literature, software must be made available to editors and reviewers. We strongly encourage code deposition in a community repository (e.g. GitHub). See the Nature Portfolio guidelines for submitting code & software for further information.

March 2021

Data

Policy information about availability of data

All manuscripts must include a data availability statement. This statement should provide the following information, where applicable:

- Accession codes, unique identifiers, or web links for publicly available datasets
- A description of any restrictions on data availability
- For clinical datasets or third party data, please ensure that the statement adheres to our policy

METSIM adipose array data are available from in the Gene Expression Omnibus database under accession code GSE70353 (https://www.ncbi.nlm.nih.gov/geo/query/acc.cgi?acc=GSE70353). The mouse RNA-seq results from eWAT of HFD-fed ParkinAdi vs. Control f/f mice and AAV8-Nqo1 vs. AAV8-Control injected eWAT have been deposited in the Gene Expression Omnibus database under accession code GSE207496 (https://www.ncbi.nlm.nih.gov/geo/query/acc.cgi?acc=GSE207496). Human PARK2 gene (encodes Parkin protein) association is available from PhenoScanner v2 (http://www.phenoscanner.medschl.cam.ac.uk). The authors declare that all data supporting the findings of this study are available within the paper and its Supplementary Information files. Source data are provided as a Source Data file.

Human research participants

Policy information about studies involving human research participants and Sex and Gender in Research.

Reporting on sex and gender	Genetic association and gene expression analyses were conducted on data collected from the metabolic syndrome in men (METSIM) study as previously described. No new human samples were acquired for the generation of this manuscript.
Population characteristics	n/a
Recruitment	n/a
Ethics oversight	n/a

Note that full information on the approval of the study protocol must also be provided in the manuscript.

Field-specific reporting

Please select the one below that is the best fit for your research. If you are not sure, read the appropriate sections before making your selection.

☒ Life sciences ☐ Behavioural & social sciences ☐ Ecological, evolutionary & environmental sciences

For a reference copy of the document with all sections, see nature.com/documents/nr-reporting-summary-flat.pdf

Life sciences study design

All studies must disclose on these points even when the disclosure is negative.

Sample size	For in vivo studies, sample size were determined based upon previous experience (PMID: 32759275; PMID: 27075628). For in vitro studies, a minimum of 3 biological replicates was used for all experiments (PMID: 29378845).
Data exclusions	Samples were excluded if the Ct values were not detectable by quantitative RT-PCR. Animals were excluded from study if disease was discovered at any point during the study or at time of necropsy.
Replication	Key experiments were independently reproduced. In vivo studies (HFD-fed Control vs. ParkinAdi mice and AAV8-Control/Nqo1 injection) were replicated in two independent cohorts. In vitro, at least 3 biological replicates were included in each experiment. The phenotypic validity of our cell lines were confirmed by monitoring morphology and gene expression with each experiment. Mycoplasma contamination was examined using commercially available PCR kits every 3-6 months (Sigma-Aldrich).
Randomization	Mice were assigned to groups based on matching for body weight and blood glucose. For all in vitro studies, cells were randomaly allocated into groups.
Blinding	Gene analysis in differentiated adipocytes, indirect calorimetry, and AAV8-Control/Nqo1 injection were performed blindly. All imaging and quantification of staining was performed on blinded samples, with data compiled at the end of the analysis.

Reporting for specific materials, systems and methods

We require information from authors about some types of materials, experimental systems and methods used in many studies. Here, indicate whether each material, system or method listed is relevant to your study. If you are not sure if a list item applies to your research, read the appropriate section before selecting a response.

Materials & experimental systems

n/a	Involved in the study
☐	☒ Antibodies
☐	☒ Eukaryotic cell lines
☒	☐ Palaeontology and archaeology
☐	☒ Animals and other organisms
☒	☐ Clinical data
☒	☐ Dual use research of concern

Methods

n/a	Involved in the study
☒	☐ ChIP-seq
☒	☐ Flow cytometry
☒	☐ MRI-based neuroimaging

Antibodies

Antibodies used

Parkin (Cell Signaling #2132, 1:1000), Pink1 (Cayman Chemicals, #10006283, 1:1000), p62 (Progen Biotechnik GmbH, #03-GP62-C, 1:1000), actin (Santa Cruz Biotechnology, sc-47778, 1:1000), Lc3b (Novus, NB100, 1:1000), GAPDH (Invitrogen, AM4300, 1:10,000), F4/80 (Santa Cruz Biotechnology, sc-377009, 1:100), Pgc1α (EMD Millipore, AB3242, 1:1000; Abcam, ab191838, 1:1000; and Abcam, ab106814, 1:1000), Hsp90 (Cell Signaling #4877, 1:1000), OXPHOS (Abcam, ab110413, 1:1000), Nqo1 (Novus, NB200-209, 1:1000; Abcam, ab80588, 1:1000), p53 (Santa Cruz Biotechnology, sc-126, 1:1000), Mono- and polyubiquitinylated conjugates recombinant monoclonal antibody (ENZO lifeSCIENCES, ENZ-ABS840, Clone UBCJ2, 1:1000), Paris (Abcam, ab130867, 1:1000), Hmgb1 (Abcam, ab18256, 1:1000), α-Tubulin (Santa Cruz Biotechnology, sc-5286, 1:1000), Perilipin 1 (Cell Signaling, #9349, 1:100), AlexaFluor 488 goat anti rabbit IgG (Invitrogen, A11088, 1:1000), or AlexaFluor 568 goat anti mouse IgG (Invitrogen, A11004, 1:1000).

Validation

All antibodies were validated by the suppliers. The respective validation data are available on the manufacturer's website. To find the right band for Pgc1α or Nqo1, two or three antibodies of each were tested using the same samples.

Eukaryotic cell lines

Policy information about cell lines and Sex and Gender in Research

Cell line source(s)

3T3-L1, CL-173, ATCC; 10T1/2, CCL-226, ATCC; Primary human subcutaneous pre-adipocytes, PCS-210-010, ATCC

Authentication

Cells were validated by differentiating to adipocytes.

Mycoplasma contamination

Cells were tested negative for mycoplasma contamination.

Commonly misidentified lines
(See ICLAC register)

No commonly misidentified lines were used.

Animals and other research organisms

Policy information about studies involving animals; ARRIVE guidelines recommended for reporting animal research, and Sex and Gender in Research

Laboratory animals

All mouse experimentation and animal care were approved by the University of California, Los Angeles Institutional Animal Care and Use Committee (IACUC). Mice were maintained on a 12-h light/dark cycle from 6 am to 6 pm at ambient temperature (~72F) with controlled humidity (~45%) in pathogen-free conditions. All mice are in C57BL/6J background about 4-6 months old. Parkin floxed mice (a gift from Ted Dawson) were crossed with adiponectin Cre mice (The Jackson Laboratory, #010803) or Ucp1 Cre mice (The Jackson Laboratory, #024670) to generate animals with Parkin deletion in adipose tissue specifically (ParkinAdi) or in brown adipose tissue separately (ParkinBAT). Whole-body parkin null mice were obtained from the Jackson Laboratory (#006582).
49 strains of mice (About 4 months old) in exercise HMDP study were listed in source data. These are 129X1/SvJ; A/J; AKR/J; AXB12/PgnJ; AXB19/PgnJ; AXB5/PgnJ; BALB/c; BTBR T<+> tf/J; BXA1/PgnJ; BXD1/TyJ; BXD11/TyJ; BXD12/TyJ; BXD14/TyJ; BXD16/TyJ; BXD19/TyJ; BXD22/TyJ; BXD24/TyJ-Cep290<rd16>/J; BXD27/TyJ; BXD28/TyJ; BXD31/TyJ; BXD33/TyJ; BXD34/TyJ; BXD38/TyJ; BXD39/TyJ; BXD44/RwwJ; BXD50/RwwJ; BXD56/RwwJ; BXD66/RwwJ; BXD70/RwwJ; BXD71/RwwJ; BXD79/RwwJ; C3H/HeJ; C57BL/6J; C57BLKS/J; C57L/J; CBA/J; CXB7/ByJ; DBA/2J; FVB/NJ; I/LnJ; LP/J; NOD/ShiLtJ; NZB/BlNJ; NZW/LacJ; PL/J; RIIIS/J; SEA/GnJ; SM/J; SWR/J

Wild animals

Study did not include wild animals.

Reporting on sex

We used both female and male whole-body Parkin knockout mice in this manuscript. We used male ParkinAdi and ParkinBAT mice for the study.

Field-collected samples

Studies did not involve samples collected from the field.

Ethics oversight

The University of California, Los Angeles Institutional Animal Care and Use Committee approved this study. All animal care, maintenance, surgeries, and euthanasia were conducted in accordance with this Institutional Animal Care and Use Committee and the National Institutes of Health.

Note that full information on the approval of the study protocol must also be provided in the manuscript.
